# Epidemiologic and import risk analysis of Peste des petits ruminants between 2010 and 2018 in India

**DOI:** 10.1186/s12917-022-03507-x

**Published:** 2022-11-29

**Authors:** Shuwen Zhang, Ruirui Liang, Qiaoling Yang, Yunfeng Yang, Songyin Qiu, Hui Zhang, Xiaosheng Qu, Qin Chen, Bing Niu

**Affiliations:** 1 School of Life Sciences, Shanghai University, Shanghai, 200444 People’s Republic of China; 2 School of Environmental and Chemical Engineering, Shanghai University, Shanghai, 200444 People’s Republic of China; 3 Chinese Academy of Inspection and Quarantine, Beijing, 100176 People’s Republic of China; 4 National Engineering Laboratory of Southwest Endangered Medicinal Resources Development, Guangxi Botanical Garden of Medicinal Plants, Nanning, 530023 China

**Keywords:** Peste des petits ruminants (PPR), Spatiotemporal analysis, Transmission dynamics, Scenario tree

## Abstract

**Background:**

Peste des petits ruminants (PPR) is a serious disease that affects goats, sheep and other small ruminants. As one of the earliest and most serious countries, PPR has seriously threatened India's animal husbandry economy.

**Results:**

In this study, the spatiotemporal characteristics of the PPR in India outbreaks were analyzed. Between 2010 and 2018, the epidemic in India broke out all over the country in a cluster distribution. Epidemic clusters in northern and southern India are at higher risk, and the outbreak time of PPR has significant seasonality. The results of the analysis of the development and transmission of PPR under the natural infection conditions showed that the PPR outbreak in India reached a peak within 15 days. Finally, the quantitative risk analysis results based on scenario tree show showed that the average probability of infecting PPRV in live sheep exported from India was 1.45 × 10^–4^.

**Conclusions:**

This study analyzed the prevalence of PPR in India. The analysis of transmission dynamics on the development of the epidemic provides a reference for the prevention and control of the epidemic. At the same time, it provides risk analysis and suggestions on trade measures for the trading countries of India.

**Supplementary Information:**

The online version contains supplementary material available at 10.1186/s12917-022-03507-x.

## Background

Peste des Petits Ruminants (PPR) is an acute, contagious animal disease caused by the Peste des Petits Ruminants Virus (PPRV) [1]. Goats, sheep and other small ruminants are susceptible to PPR. The outbreak of PPR causes great economic losses to livestock. It is estimated that the global economic impact of PPR is between US $1.4 billion and US $2.1 billion [2]. PPR was first discovered in 1942 in Cote d'Ivoire, West Africa. Before 1979, PPR outbreaks mainly occurred in the neighboring countries of Cote d'Ivoire, such as Ghana, Benin and Nigeria [3–5]. After that, the PPR epidemic began to spread from West Africa to East Africa. In 1987, PPR reached the northern part of India in Asia and spread to the Arabian Peninsula, the Middle East and other parts of India [6]. In 1999, initials PPR spread to Turkey in Europe [7]. So far, the PPR epidemic has spread to Europe, Asia and Africa, more than 70 countries have found PPR, among which Asia and Africa have the largest number of outbreaks, involving a wide range of regions [8].

According to the statistical data of the global animal disease information system, PPR broke out in India, Bhutan, Georgia, Israel, Mongolia, Nepal, Thailand and other Asian countries from 2015 to 2020. PPR, as an animal disease that seriously affects the healthy development of the animal husbandry economy, also has an impact on animal trade. India is one of the most affected Asian countries affected by PPR [9]. The epidemic of PPR has an impact on animal health and animal trade in India, and it is reported that the annual economic losses caused by PPR epidemics ranged from US $ 2 million to US $ 18 million per year [10]. Since PPR can spread through animal trade, and India has a large number of sheep, which will lead to a greater risk of PPR infection and transmission in India. According to the statistics of the Indian Ministry of agriculture, there are about 223 million goats and sheep in India in 2019, which accounted for 41.58% of the total animal population in India. India is at risk of PPR infection because of the large number of susceptible populations. Therefore, this paper chooses India as the research object to study the development and prevalence of the PPR epidemic and its impact on trade.

In terms of animal disease analysis, the spread of disease can be clearly analyzed through the dynamic interaction of time and space of disease outbreak [11]. Transmission dynamics models are often used to further understand the evolution and spread of diseases. Among them, the basic reproduction number (R_0_) is used to evaluate the transmission ability and risk of animal diseases [12]. PPR as a global disease, trade is one of the important ways of disease transmission. A scenario tree can be used to analyze each link in the process of animal and animal products trade, and evaluate the risk of virus release through trade. Isoda et al. evaluated the risk of introducing animal diseases due to animal trade by analyzing control measures such as testing and vaccination of imported animals [13]. In addition, scenario tree can also be used to assess the risk of disease occurrence in areas where animal trade is restricted. Spatiotemporal analysis, transmission dynamics and scenario tree are commonly used to analyze the animal epidemic situation.

**Fig. 1:**
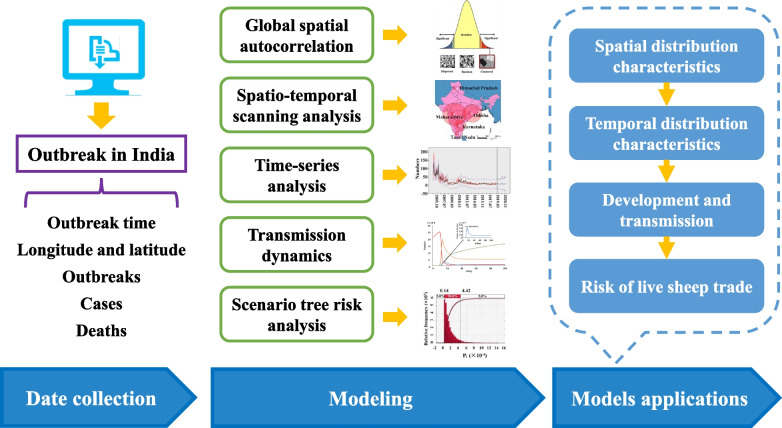
Flow chart of PPR epidemic analysis in India

In this paper, we analyzed the spatial and temporal distribution characteristics of the PPR epidemic in India. At the same time, we built a time-series analysis model to predict the occurrence of PPR in India. Secondly, according to the transmission characteristics of PPR, SEIR (Susceptible-Exposed-Infectious-Removed) transmission dynamics model was constructed to analyze the development process of PPR in India and reveal the epidemic law. Finally, the quantitative risk analysis of the scenario tree was used to evaluate the risk of exporting PPRV-infected sheep from India (Fig. 1).

## Results

### Temporal and spatial characteristics of PPR in India

**Fig. 2:**
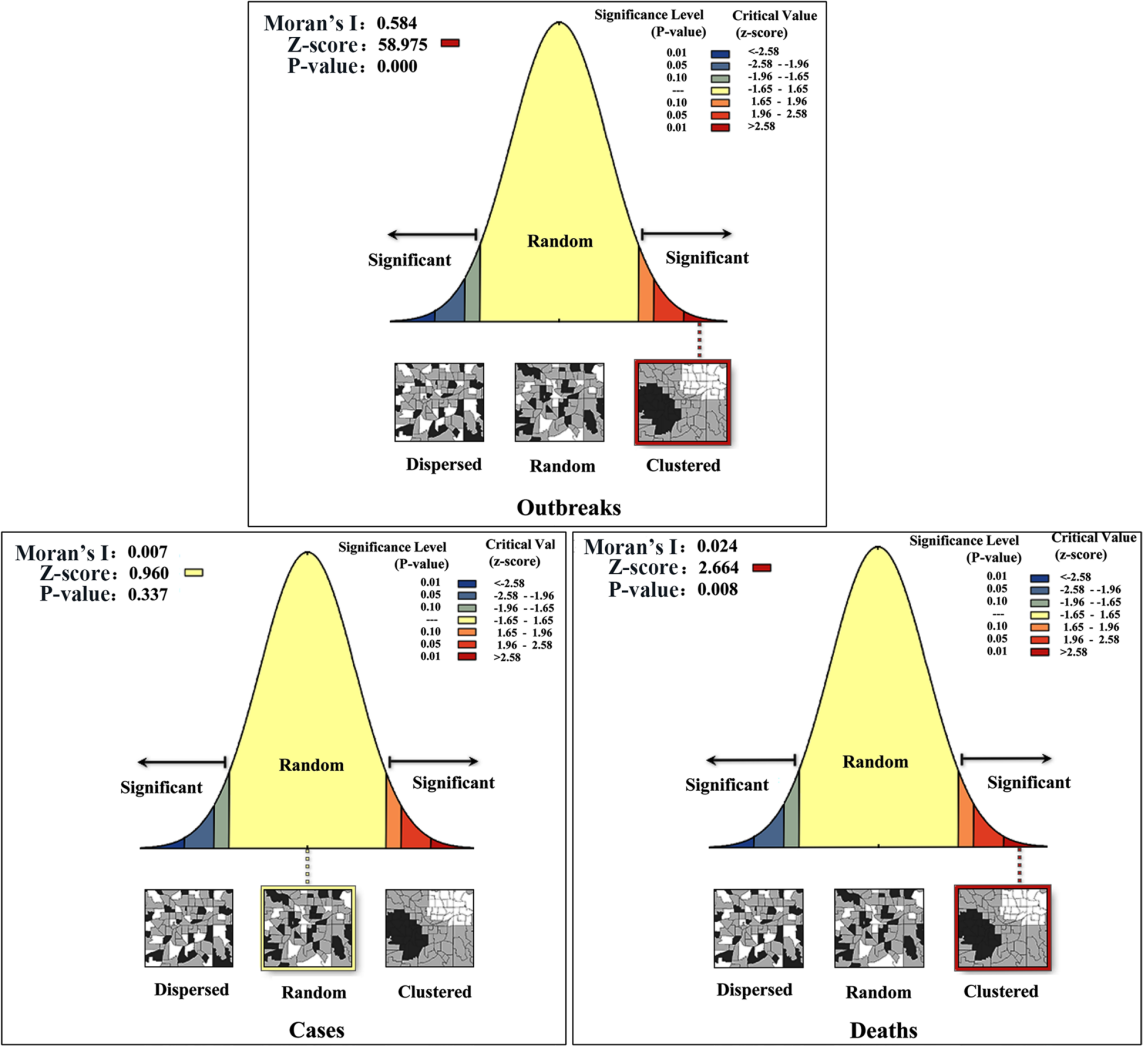
Significance test of global spatial autocorrelation of PPR in India from 2010 to 2018 Note: The data of PPR outbreak in India was collected from WOAH (https://www.woah.org/) and EMPRES-i (http://empres-i.fao.org/eipws3g/#h=0) and the spatial correlation was created by ArcGIS software (version 10.1, ESRI Inc.; Redlands, CA, USA)

Based on the spatial clustering analysis of outbreaks, cases and deaths of the PPR epidemic in India from 2010 to 2018, the results (Fig. 2) showed that the number of outbreaks and deaths of the PPR epidemic in India are distributed in an aggregated spatial pattern. However, in the spatial clustering analysis of PPR cases in India, we find that the Z-score was 0.96 (*P* > 0.1), indicated that there was no significant difference between the distribution of PPR cases in India and showing a spatial random distribution.

**Fig. 3:**
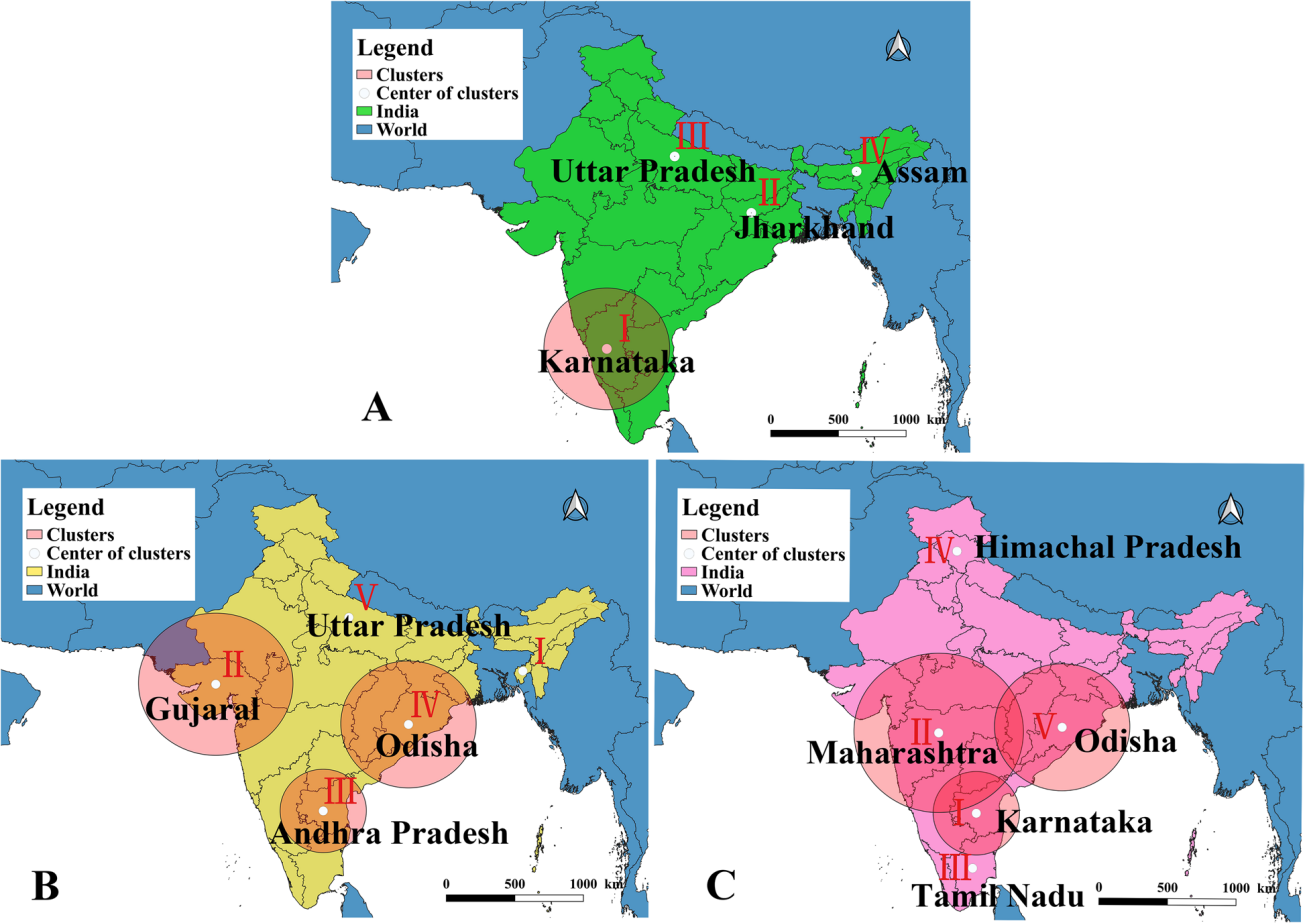
Statistical analysis of spatio-temporal scanning of PPR epidemics in India from 2010 to 2018 Note **A** Distribution of PPR outbreaks in India from 2010 to 2018, **B** Distribution of PPR cases in India from 2010 to 2018, **C** Distribution of PPR deaths in India from 2010 to 2018

According to the spatiotemporal scanning statistics of PPR outbreaks in India, four clustering areas were obtained (Fig. 3A and Table 1). Among them, Cluster I was located in Karnataka state in the south of India, which had the widest coverage (cluster radius was 453.3 km) and the highest number of outbreaks, but the risk of Cluster I was low. There was a difference between the actual and expected values in Clusters II and IV, which indicated that the high potential risk value of PPR outbreak in West Bengal and Assam in Clusters II and IV. According to the spatiotemporal scanning statistics of the number of cases and the number of deaths, we obtained five clustering regions, respectively (Fig. 3B; C). The results of spatiotemporal scanning statistical analysis of the number of cases showed that the Clusters II and IV at low potential risk. However, the smallest Cluster I and V have high potential risk. The results of spatiotemporal scanning statistical analysis based on the number of deaths showed that Cluster I, II and V gathering area was the largest with low risk. In contrary, Clusters III and IV had a high risk.

**Table 1:** Analysis of spatio-temporal scanning of PPR epidemics in India (2010 – 2018)

Title	Location IDs	Radius	Number of cases	Expected cases	RR	LLR
Outbreaks	I	453.30	121	52.37	2.31	34.82
	II	0	11	0.55	19.85	22.47
	III	0	19	2.5	7.61	22.16
	IV	0	7	0.25	27.79	16.54
Cases	I	0	2172	89.04	24.39	4893.21
	II	522.56	6771	1734.03	3.9	4420.94
	III	306.66	6963	1851.92	3.76	4352.67
	IV	466.29	10,496	4671.35	2.25	3005.64
	V	0	1000	33.66	29.71	2433.25
Deaths	I	306.66	1808	503.53	3.59	1072.36
	II	585.20	2467	972.02	2.54	892.60
	III	0	632	78.54	8.05	775.68
	IV	0	738	122.3	6.03	724.80
	V	466.29	1640	582.4	2.82	683.37

*LLR* Log Likelihood Ratio, *RR* Relative risk

**Fig. 4:**
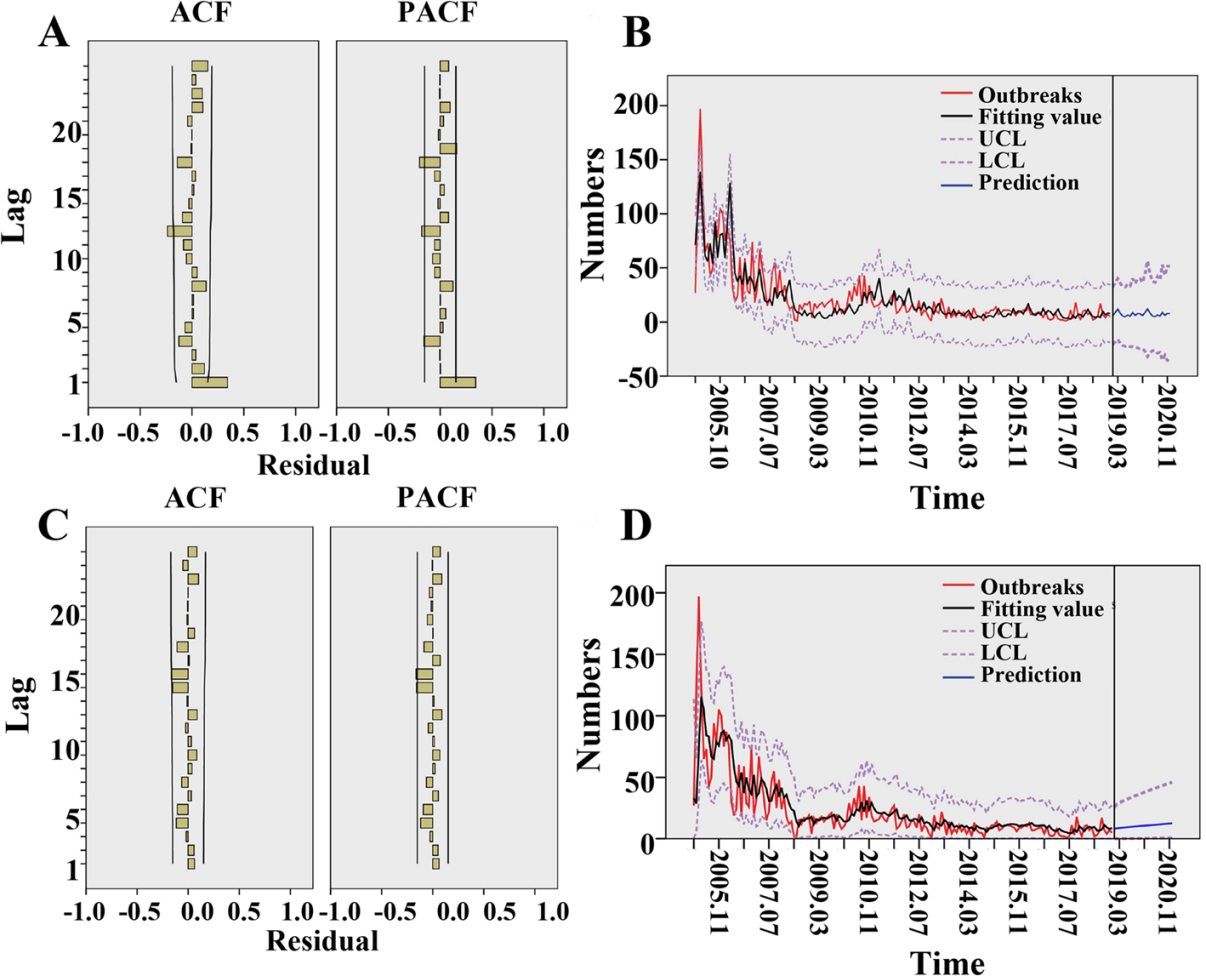
Modeling results of winters multiplication model and ARIMA model Note **A** and **B** are the results of winters multiplication; **C** and **D** are the results of the ARIMA model; **A** and **C** are partial correlation analysis; **B** and **D** are the prediction curves

The PPR epidemic in India from January 2005 to December 2018 was analyzed in time series. The outbreak in India has seasonal characteristics, and an additional file shows this in more detail (see Additional file 1,Fig. S1A). According to the results of seasonal decomposition (see Additional file 1, Fig. S1B and Table 2), the seasonal factor values of January, February, March, September, November and December were positive, indicating that these months were peaks of the PPR epidemic in India. The outbreak data of PPR in India was a non-stationary series and showed the seasonal effect of the cycle, to reduce the impact of time on the model. The BIC of winters multiplication model and Autoregressive Integrated Moving Average model (ARIMA) were 5.297 and 5.820 respectively (see Additional file 1, Table S1). Through the residual analysis (the difference between the observed value and the predicted value), it was found that most of the Autocorrelation Function (ACF) and Partial Autocorrelation Function (PACF) were in respective ranges, indicating that the datas were reliable Fig. 4A and C). According to prediction models, the number of outbreaks in India in 2020 was predicted. Within the 95% confidence interval, the prediction results showed that PPR outbreaks may continue in India in 2020 (Fig. 4B, D and Table S2).

**Table 2:** Seasonal factor values of PPR outbreaks from 2005 to 2018

Month	Seasonal factor values	Month	Seasonal factor values
1	0.734	7	-0.532
2	0.493	8	-4.058
3	2.878	9	5.202
4	-0.837	10	-0.673
5	-3.132	11	3.506
6	-5.744	12	2.163

### Transmission dynamics model of PPR in India

**Fig. 5:**
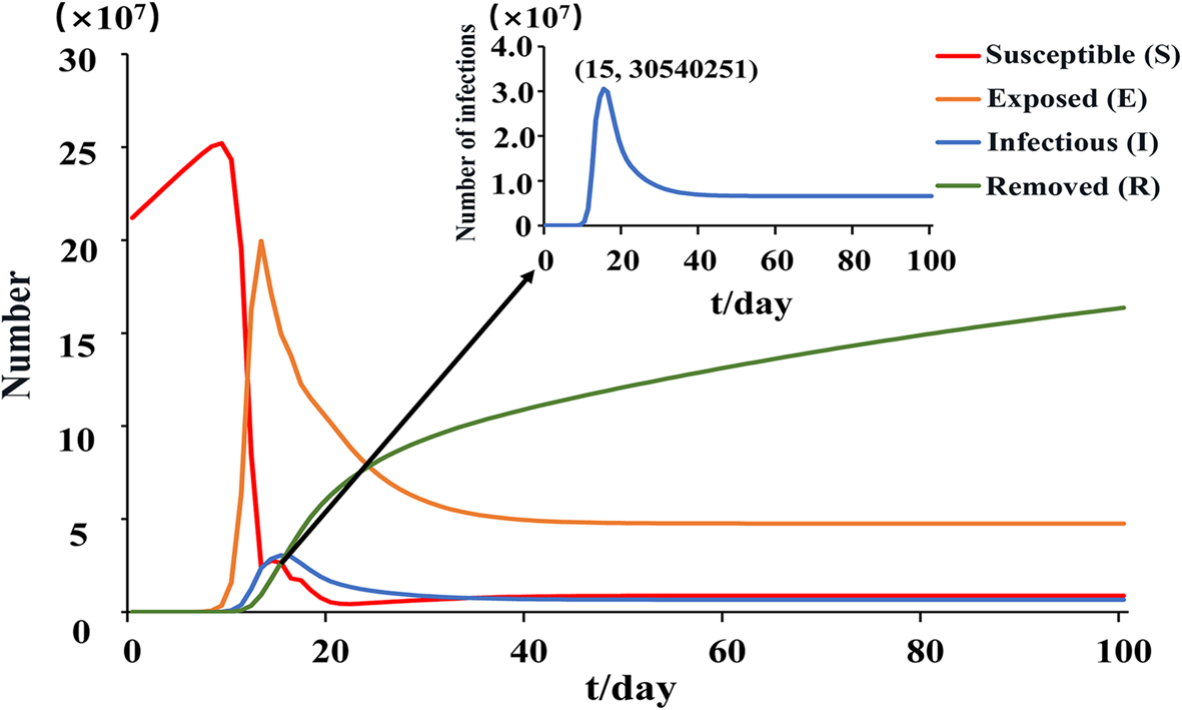
Simulated transmission of petite-ruminant disease in India

The initial conditions of India SEIR (Susceptible-Exposed-Infectious-Removed) model were set as follows: S (0) = 211,971,425, E (0) = 0, I (0) = 1, R (0) = 0 (Table 3). S (0), E (0), I (0) and R (0) represent the susceptible number of animals, number of exposed animals, number of infected animals and cure number of animals. The results showed that the number of animals infected with PPR increased first and then decreased under the natural infection conditions without control measures in India (Fig. 5). The number of infected animals reaches the peak around 15 days, and about 3.1 × 10^7^ sheep were infected with PPR.

**Table 3:** Parameter description of India SEIR model

Notation	Definition	Values	Source
N_i_	Total population	211,971,425	India-BAHS
n_i_	Total population input in the model	7,222,500	National Bureau of Statistics
u_i_	Natural mortality	0.01%	/
P_i_	Mortality	65.30%	WOAH(death/case)
β_i_	Exposure infection rate = c*h	1.24 × 10^–7^	[14]
c_i_	Number of exposure/N	66/211971425	/
h_i_	Probability of illness	39.70%	[15]
$\omega$_i_	Reciprocal of the disease incubation period	1/T	/
T	Incubation period	7	WOAH/FAO
γ_i_	The recovery rate of infected patients	34.70%	1-Pi

**Fig. 6:**
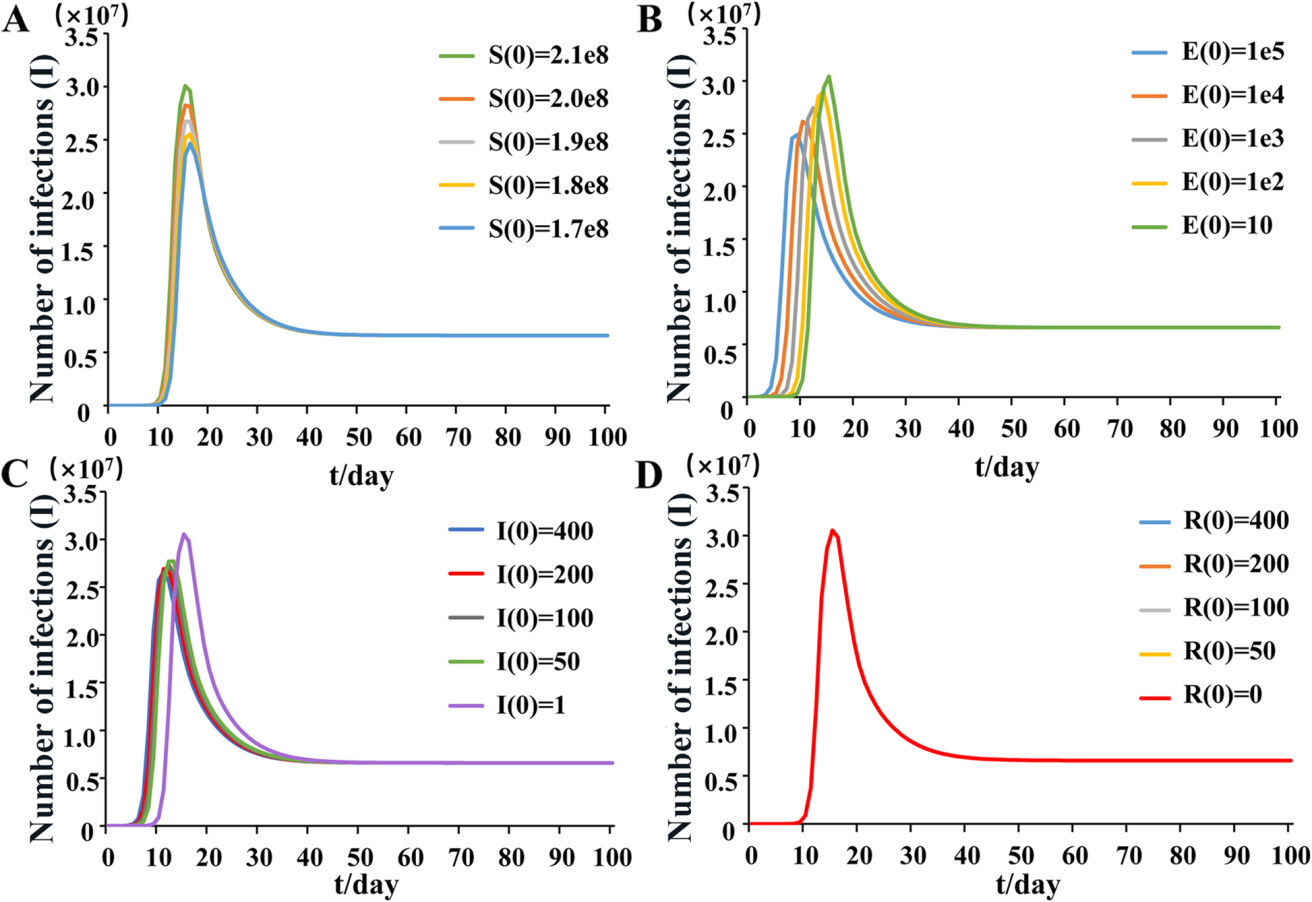
Impact of changes in initial conditions on the number of infected (I) in SEIR model of India Note **A** S(0); **B** E(0); **C** I(0); **D** R(0)

By changing the initial conditions S (0), E (0), I (0) and R (0) of the model, the relationship of the number of Indian sheep infected with PPR virus (I) with time (t) was analyzed (Fig. 6). With the increase of the initial susceptible animal number (S (0)), the peak time of infection number (I) gradually advanced (Fig. 6A). When S (0) was constant, with the increase of the number of exposed animals (E (0)) and the number of infected animals (I (0)) in the initial incubation period, the time to reach the peak of the number of infected animals gradually decreases, that is, the infection rate of PPR is accelerated (Fig. 6B; C). In Fig. 6D, it was shown that the initial value of R0 does not affect Infectious (I). The transmission speed of PPR was related to the number of susceptible animals, infection and exposure.

**Fig. 7:**
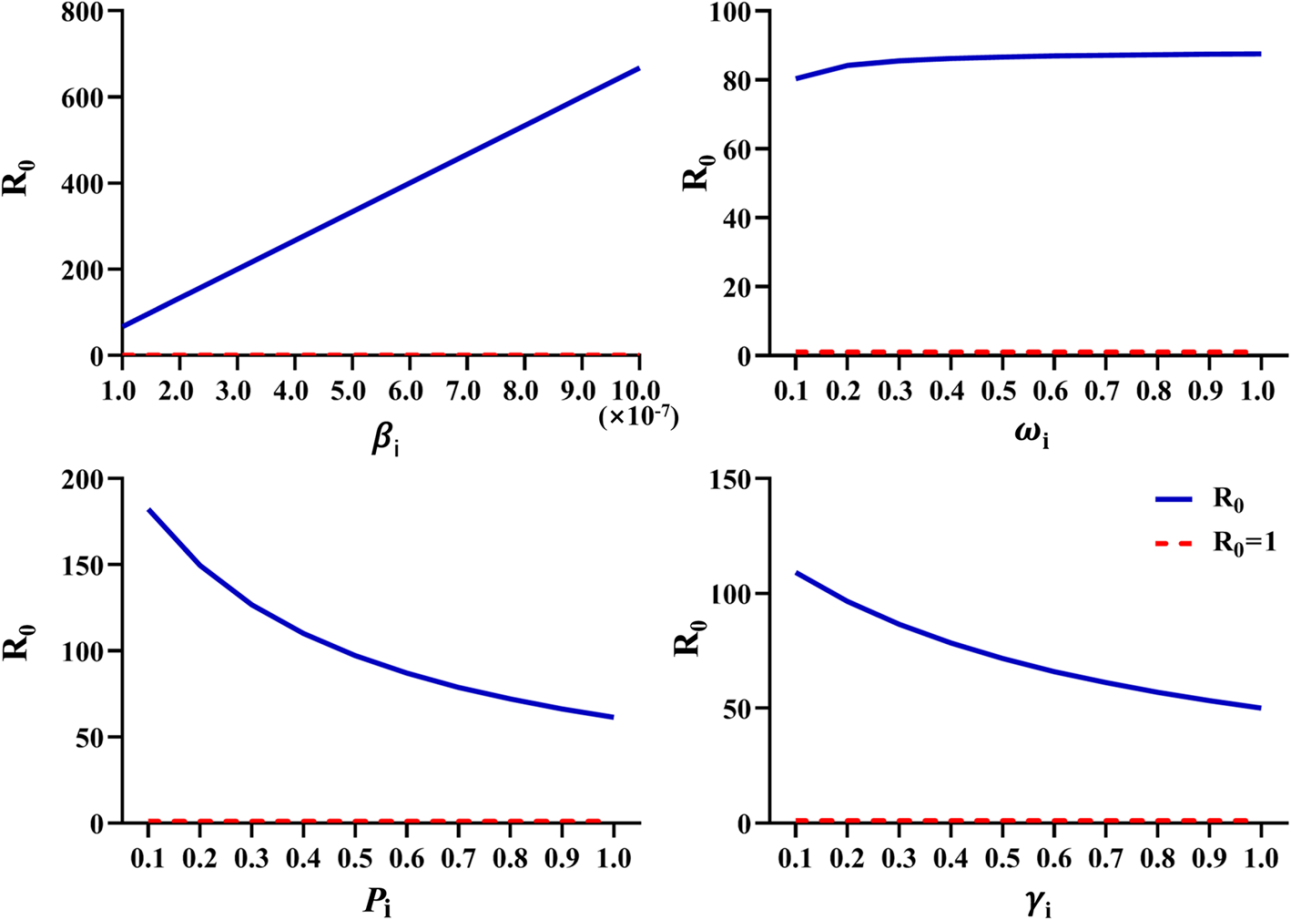
Influence of different parameters on the basic regenerative number (R_0_) in SEIR model of India Note β_i_: Exposure infection rate; ω_i_: The infection intensity in incubation period; γ_i_: Recovery rate of infected patients; p_i_: Mortality

The value of R_0_ showed the transmission ability of the disease, and the influence of different parameters on R_0_ was evaluated (Fig. 7). R_0_ increases with the increase of the rate of contact infection (*β*_*i*_) and the infection intensity in the incubation period (ω_i_) had little effect on R_0_. Under the assumption that the dead animals were not infectious after treatment, with the increase of mortality (*Pi*) and recovery cure rate (γi), R_0_ gradually decreased, that is, the transmission rate of the disease gradually decreased.

### Quantitative risk analysis of PPR through trade

**Fig. 8:**
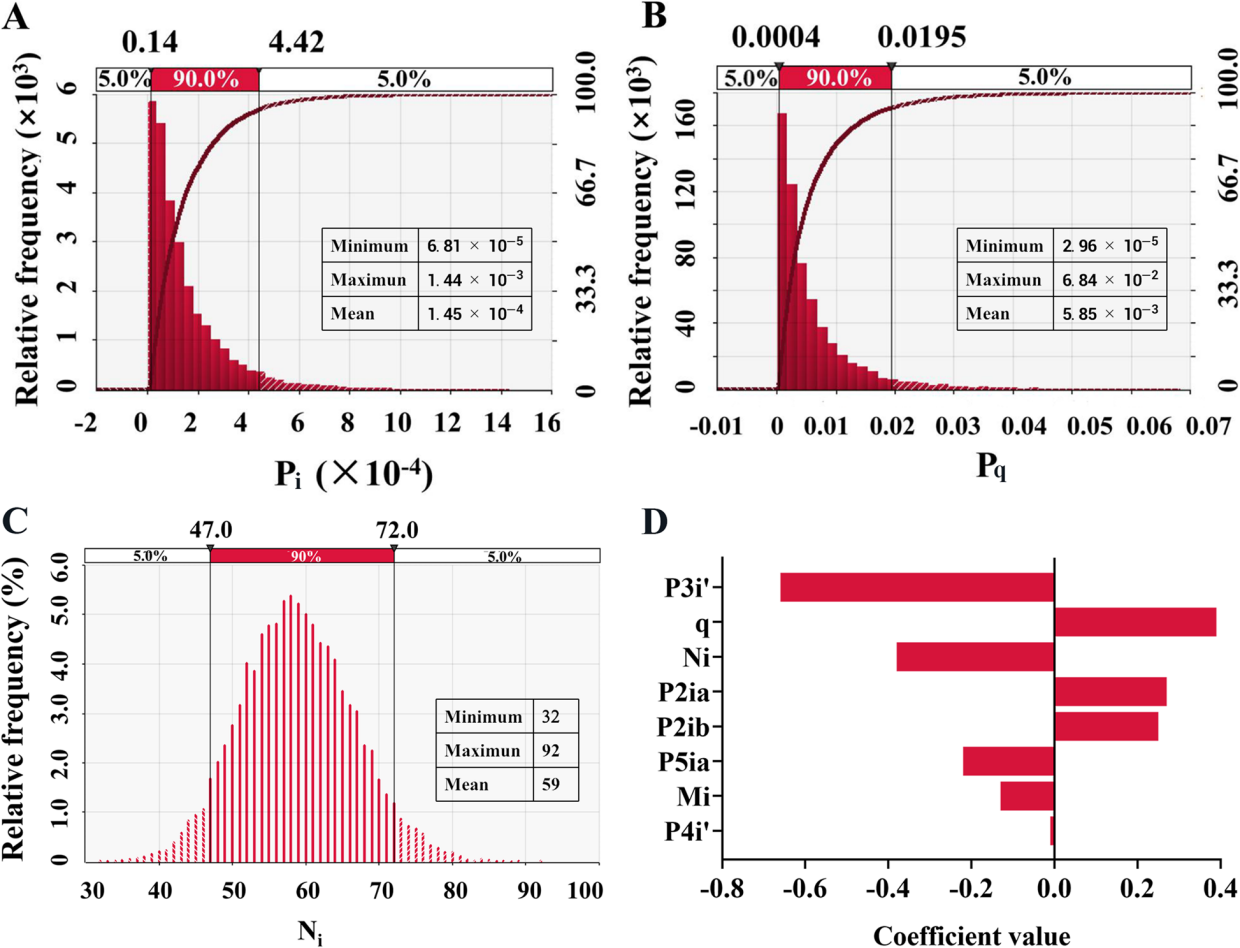
Output results of scenario tree quantitative risk analysis model in India Note **A** Probability of PPRV infection in live sheep exported from India (P_i_); **B** Probability of at least one sheep infected with PPRV exported from India (P_q_); **C** Number of infected sheep exported per year (N_y_); **D** Sensitivity analysis of correlation coefficient of India scenario tree

Monte Carlo simulations were conducted to obtain the risk output of the model (Fig. 8). The results showed that the average probability of PPRV infection in live sheep exported from India (Pi) was 1.45 × 10^–4^ (Fig. 8A). The average probability of infection of at least one animal in the export of live sheep (Pq) was 5.85 × 10^–3^ (Fig. 8B). India exports an average of 300,000 sheep a year. After binomial conversion, the average number of sheep infected with PPRV exported each year was 59 (Minimum was 32 and Maximum was 92) (Fig. 8C). The sensitivity of ELISA (P3i’), the export volume of live sheep in India (q) and the Probability of infected animals undetected in quarantine (P6i) had the greatest impact on the risk value of PPRV output (Fig. 8D).

As shown in Table 4, we further analyzed the impact of the P3i node on the model output risk results. Ignoring the clinical inspection (P3i), the average probability of PPRV infection in live sheep exported from India (Pi) was 0.02, and the probability of at least one sheep infected with PPRV exported from India (Pq) was 0.49. The number of sheep infected with PPRV was 8177 per year. After enhancing the P3i node, the average probability of PPRV infection in live sheep exported from India was 2.04 × 10^–6^. Therefore, strengthening clinical examination (P3i) can reduce the risk value.

**Table 4:** India's PPRV risk exports through the live animal trade

Node settings	Output	Minimum	Maximum	Mean
Ignore P3i clinical inspection	Pi	4.03 × 10^–4^	0.14	0.02
	Pq	1.46 × 10^–2^	0.99	0.49
	Ni	7830	8550	8177
Enhance P3i clinical inspection	Pi	4.14 × 10^–8^	1.13 × 10^–5^	2.04 × 10^–6^
	Pq	1.75 × 10^–6^	8.39 × 10^–4^	8.39 × 10^–5^
	Ni	0	1	6

## Discussion

Peste des petits ruminants (PPR) is a cross-border viral disease, threatening the health of about 1.74 billion goats and sheep, which has a huge impact on the animal husbandry economy. In 2015, the international community set the goal of eliminating PPR by 2030 [16]. With the increase of global sheep density and trade in animals and animal products, the possibility of a PPR outbreak also increases. Understanding the transmission route of PPR and the occurrence, development and evolution of PPR, and formulating scientific and effective prevention and control measures are the key to control the spread of PPR.

PPR has been discovered for the first time in Tamil Nadu, India in 1987. Sheep and goats play a vital role in the socio-economy of rural families in India. These animals are important economic sources [17]. In this study, after spatiotemporal analysis, the number of outbreaks and deaths of PPR in India showed an aggregated spatial distribution, but the number of cases of PPR was scattered and showed a random distribution so it was difficult to control and eradicate the PPR epidemic in India. Our results of spatiotemporal scanning analysis showed that Western and northern India has a higher risk of outbreaks and cases, and southeast India has a higher risk of death. According to the survey results of Balamurugan et al. [9], high density areas of sheep and goats are distributed in the southeast and the mortality of PPR in eastern India was higher than that in other regions. In addition, according to the time series analysis of the PPR epidemic trend in India, the PPR outbreak in India has obvious seasonal characteristics. This may be affected by the mode of production, climate and geographical location of animal husbandry [18]. Our study found that PPR outbreaks in India often occurred in wet periods (March and September) or cold and drought periods (January, February and December), which was consistent with the results of Singh 's study [19]. From December to February, the weather is cold and dry, the food and grass are insufficient in winter, the nutrition of susceptible animals is insufficient and the immunity is reduced. The severe cold weather and flocking of livestock have promoted the spread of PPRV. In addition, drinking water of susceptible animals in rainy season also indirectly increased the spread of the virus [9]. In India, sheep and goats are mostly raised by nomadic people. During the lambing season, free grazing, or looking for pasture to trade during the poor pasture period, the migration process increases the chance of PPRV infection. The prediction results of winters multiplication model and ARIMA model constructed in this study showed that PPR epidemic may continue to break out in India.

Through the understanding of the PPR transmission pathway and the evolution law of occurrence and development, it provides a theoretical basis for the development of scientific and effective prevention and control measures. The formulation of prevention and control measures is also an urgent task to control the spread of PPR. Therefore, in this study, according to the transmission characteristics of the PPR epidemic, SEIR (Sustainable-exposed-infectious-removed) transmission dynamics models were constructed in India. Under natural infection conditions without control measures, India reached the peak of the outbreak in about 15 days. The basic reproduction number (R_0_) value shows the strength of the disease transmission ability. The R_0_ of India was greater than one, which indicated that the PPR epidemic was still in a state of infection and would not die out naturally.In addition, the effects of different parameters on R_0_ were evaluated. The results showed that R_0_ increased with the increase of infection rate. With the increase of mortality and recovery cure rate, R_0_ gradually decreased, which was the hypothesis that the dead animals were not infectious after treatment. But in reality, dead animal carcasses are infectious, so control measures should be taken to prevent the spread of the disease. In addition, the spread of the disease can be controlled by finding the infection symptoms in time and taking scientific and effective isolation treatment measures to reduce the contact infection rate and improve the cure rate. Since 2002, in order to control the PPR epidemic, some Indian states have implemented centralized vaccination to curb the spread of the epidemic [20]. In 2012 and 2014, some states began to implement mass vaccination through national control plans (PPR-CP, http://www.dahd.nic.in). Although vaccination has been implemented on a large scale, factors such as imperfect surveillance systems and untimely post-vaccination monitoring and evaluation can still lead to PPR outbreaks [21]. At present, there are few systematic epidemiological investigations and monitoring in Indian [19, 22]. Disease reporting, epidemiology, disease surveillance, diagnostic support, and vaccination of susceptible populations can effectively control and eliminate PPR epidemics.

The risk of PPR transmission from India live sheep showed that Indian has a high risk of potential PPR export to other countries. Combine the results of Spatio-temporal analysis and time series analysis, southern India has a higher mortality rate, and the outbreak of PPR is seasonal in India. We speculate that if live sheep are exported from southern India and the export time is in rainy season or cold and dry season, there is a higher risk of PPR in exported live sheep [20]. Through the sensitivity analysis of the quantitative risk assessment model of the live sheep trade in India, the results showed that the sensitivity and specificity of ELISA in clinical examination had the greatest impact on the risk value of PPRV output. Clinical examination can effectively crack down on smuggling and other illegal activities. Therefore, by enhancing the sensitivity and specificity of ELISA in clinical examination, the risk of PPRV output could be reduced [23]. Increasing export inspection and quarantine can strengthen the clinical detection ability of epidemic diseases, but if the biological safety is insufficient, inspection and quarantine will also increase the risk of infection. According to UN Database (comtrade.un.org), India exports hundreds of thousands of live sheep every year. Asian countries such as the United Arab Emirates, Nepal and Oman are major importers. It is worth noting that PPR outbreaks have occurred in United Arab Emirates, Nepal and Oman [15, 24]. Moreover, Shaila et al. showed that the strains in Oman were similar to those in southern India, and speculated that the genetic relationship between strains may reflect the trade of living animals [25].India has an extended border with many border countries such as Bangladesh, Pakistan and China. The phylogenetic tree analysis of PPR virus showed that PPR strains in Asia were closely related, and China, India, Bangladesh and Pakistan were all located in the same lineage (lineage IV) [25–27]. According to FAO data, PPR has been reported in 23 provinces in China, and a total of 29 incidents of PPR epidemics have been reported in China from 2016 to 2021. Rahman et al. found that the median of PPR infection per 10,000 small ruminants in Bangladesh was 198 [28]. Abubakar et al. conducted PPR serological analysis in various regions of Pakistan and found that the prevalence rate varies greatly among regions. The prevalence rate was estimated to be higher than 50% in some regions and lower than 10% in others [29]. Asia has a vast territory and a large number of susceptible animals. Animal migration and animal trade through legal or illegal channels (such as smuggling) may increase the risk of PPR epidemic spreading to surrounding countries [30]. In addition, the seasonal migration and grazing activities of susceptible livestock groups indirectly increase the transmission risk of PPR disease. Therefore, trading partners and neighboring countries need to strengthen the monitoring of the PPR epidemiologic situation in border areas, strengthen the inspection and quarantine in the process of trade, and put an end to all illegal smuggling. At the same time, strengthen the communication and exchange of epidemic information with other countries, and make joint efforts for the global plan to eradicate PPR.

## Conclusion

The spatial characteristics of the PPR epidemic in India showed that the number of outbreaks and deaths of PPR were distributed in a clustered space, and the herds in southern India have high mortality rate of epidemic disease. Further time series analysis showed that the outbreak of PPR in India has obvious seasonality. Secondly, through the analysis of PPR development and transmission law in India, it is found that the epidemic can quickly reach the peak without external interference. Finally, the impact of the outbreak of PPR on trade was studied. The results showed that the average probability of infecting PPRV in live sheep exported from India was 1.45 × 10^–4^. The average number of sheep infected with PPRV exported from India each year was 59. If it is in high-risk areas or epidemic seasons, the export risk may be higher. This study provides a reference for understanding the epidemiology of PPR in India. At the same time, it provides PPR risk assessment and prevention and control measures for countries importing small ruminants from India.

## Materials and methods

### Data collection

The outbreak data of Peste des petits ruminants in India were collected from the global animal disease information system (EMPRES-i, https://empres-i.apps.fao.org/diseases. Filters; Disease: Peste des petits ruminants. Region: Asia, Subregion: Eastern Asia or Southern Asia, Country: China or Bangladesh or Pakistan, Diagnosis source: All Diagnosis status: All Animal type: All), World Organization for Animal Health (WOAH, https://www.woah.org/en/disease/peste-des-petits-ruminants/), the Food and Agriculture Organization (FAO, http://www.fao.org/) and National Bureau of statistics of India (https://www.mospi.gov.in/). The simulation data of related nodes of quantitative risk analysis in India were also collected, see Table 5 for specific data sources.

**Table 5:** Input variables and sources of PPRV model in live sheep exported from India

Notation	Definition	Parameterization	Values	Source
P1i	Probability of PPR infection in India	P1i = 1-e^(−t×λ)^	P1 = 1-e^(−1191×108)^	WOAH
P2i	Probability of each sheep infected with PPRV	(P2ia + P2ib)/2	/	/
P2ia	Prevalence of sheep	/	Uniform (0.0055, 0.6305)	[31]
P2ib	Prevalence of goats	/	Uniform (0.0559, 0.6211)	[32]
P3i	Possibility of undetected in the pre-shipment inspection	1-P3i’	/	/
P3i’	Sensitivity of ELISA	/	Uniform (0.8728, 0.9703)	[33]
P4i	Survival rate of sheep in transit	1-P4i’	/	/
P4i’	Mortality of sheep in transit		Pert (0.0013, 0.0086, 0.0553)	FAO
P5i	Probability of undetected PPRV infection at destination	/	P3 × (1- P5ia)	/
P5ia	The mortality rate of sick animals	/	Uniform (0.35, 0.6)	[10]
P6i	Probability of infected animals undetected in quarantine	1-(Mi × Ni)	/	/
Mi	Possibility of quarantine	/	Beta (42.11, 1.83)	[34]
Ni	Possibility of detection during quarantine	/	Beta (15.03, 2.55)	[34]
q	Export volume of live sheep in India	/	Triang (147,649, 147,756,935,999)	FAO
Pi	Probability of PPRV infection in live sheep exported from India	P1i*P2i*P3i*P4i *P5i*P6i	/	/
Pq	Probability of at least one sheep infected with PPRV exported from India	1-(1-Pi)^q^	/	/
Ny	Number of infected sheep exported per year	/	Binomial (q, Pi)	/

### Spatiotemporal analysis

#### Global spatial autocorrelation

Global spatial autocorrelation is to analyze the correlation degree between outbreaks in a certain research area [35]. The commonly used measure is the global Moran's index. When Moran's I > 0, indicates that there is a positive spatial correlation, that is, there is the same change trend between the research elements and the adjacent elements, and the spatial distribution presents a cluster distribution. Similarly, when Moran's I < 0, it indicates that there is a spatial negative correlation, showing a random distribution. When Moran's I = 0, there is no spatial correlation. At the same time, z-score and *p*-value were used to evaluate the significance of Moran's I. ArcGIS 10.1 software (http://www.esri.com/arcgis) was used for spatial autocorrelation analysis. Fixed distance zone is a spatial relationship conceptualization parameter and the conceptualization of spatial relationships parameter was Inverse Distance, distance method parameter was Euclidean Distance.

#### Spatio-temporal scanning analysis

Spatio-temporal scanning is a statistical method to analyze data based on moving scanning window with time information. In the two dimensions of time and space, a two-dimensional cylinder active window was established, and the cylinder represents the possible gathering area. The circle at the bottom of the cylinder represents the geographical area, and the height of the cylinder represents the time. By changing the radius and period, the whole cylindrical window changes with the region and time [36]. Therefore, the geographic regions and periods of all possible outbreaks were detected. Log-likelihood ratio (LLR) was calculated to evaluate whether the clustering region was included. Relative risk (RR) was used to evaluate the risk of PPR in the cluster area. In this study, SaTScan v9.6 software (https://www.satscan.org/) was used for scanning statistical analysis, the maximum radius was 50% of the total population, and the number of Monte Carlo simulations was 999 [37]. When p < 0.05, the difference of scanning results was statistically significant, and the area had aggregation characteristics. To further analyze the characteristics of PPR epidemic areas in India, we conducted spatio-temporal scanning analysis on the number of outbreaks, cases and deaths.

#### Time-series analysis

Time series analysis is a kind of analysis method based on time sequence data, which analyzed by R language 3.5.1 software. According to the law of data change and corresponding model, the future development trend of time series data was predicted [38]. The autoregressive integrated moving average model (ARIMA) was a classical time series prediction method, which was widely used because of its simple operation, wide applicability and high accuracy in the prediction of infectious diseases. ARIMA product season model is a commonly time-series model, its form was ARIMA (p, d, q)$\times$(P, D, Q), where p was the number of autoregressive terms, d was the number of nonseasonal differences needed for stationarity, q was the number of lagged forecast errors in the prediction equation, P was the number of seasonal autoregressive terms, D was the number of seasonal differences, and Q was the number of seasonal moving average terms.

The process of building the model to predict the outbreak trend of PPR in India was as follows: First, Judge whether the time series is stationary. If it was not a non-stationary time series, it was transformed into a stationary series by difference or natural logarithm. Second, the time series model was determined by analyzing the autocorrelation function (ACF) and partial autocorrelation function (PACF) of the above stationary series. Third, the Bayesian Information Criterion (BIC) value was used to evaluate the model parameters and selected the better prediction model. The model with the smallest value of BIC was selected as the prediction model. Fourth, the model prediction results were compared with the actual outbreaks. Fifth, the occurrence of the PPR epidemic in India was predicted.

### Transmission dynamics

To calculate the transmission dynamics of PPR disease, it is important to consider the outbreak characteristics of PPR, especially the PPR has an incubation period. Therefore, the following assumptions were made: First, the route of transmission was considered as direct contact without considering the difference of susceptibility among populations; Second, the susceptible animals were sheep and goats, regardless of population migration; Third, the cured animals have immunity and will not infect PPRV again; Fourth, the model animal population was divided into four types: susceptible (S), exposed (E), infectious (I) and removed (R). The number of the four types of the population at t time was S (t), E (t), I (t) and R (t), respectively; Fifth, susceptible animals (S) contacted with infected animals (I) and were transfected into exposed animals (E). The transmission rate of contact was β. During the incubation period, the exposed animals (E) will transform into the infected animal (I), which was based on the probability of transformation ω. The cure rate of an infected animal (I) was γ.

**Fig. 9:**
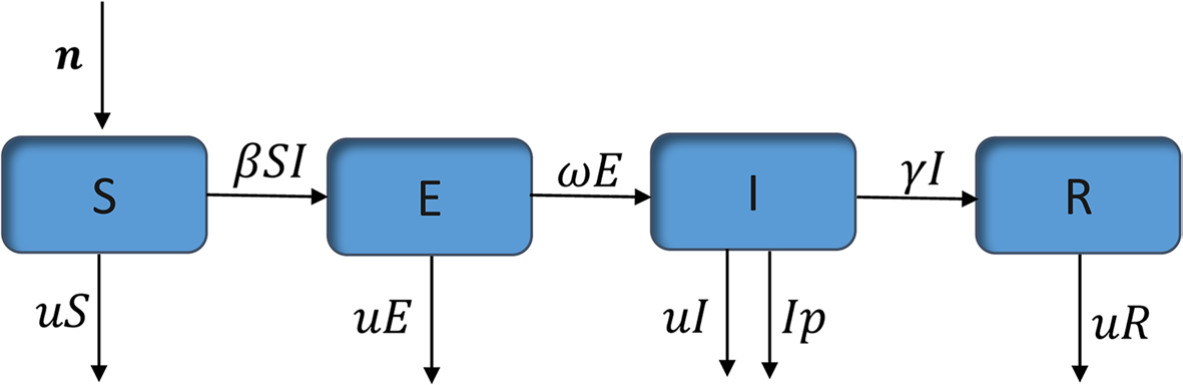
Flow Chart of SEIR (Susceptible- Exposed-Infectious-Removed) model

According to the assumptions, SEIR (Susceptible- Exposed-Infectious-Removed) model was established. According to the flow chart (Fig. 9), the differential equations of the model were as follows:

1 $$\frac{\mathrm{dS}(\mathrm{t})}{\mathrm{dt}}=n-\beta SI-uS$$

2 $$\frac{\mathrm{dE}(\mathrm{t})}{\mathrm{dt}}=\beta SI-\omega E-uE$$

3 $$\frac{\mathrm{dI}(\mathrm{t})}{\mathrm{dt}}=\omega E-\gamma I-I\left(u+p\right)$$

4 $$\frac{\mathrm{dR}(\mathrm{t})}{\mathrm{dt}}=\gamma I-uR$$

where $n$ was total population input in the model, β was exposure infection rate, $u$ was natural mortality,$\omega$ was the reciprocal of disease incubation period, γ was recovery rate of infected patients, $p$ was mortality.

The basic reproduction number, R_0_, was the most important index to measure the transmission ability of the virus. If R_0_ < 1, the infectious disease will disappear gradually. If R_0_ > 1, the infectious disease will spread exponentially and become epidemic. If R_0_ = 1, the infectious disease will become an endemic disease in the population. The larger the number of R_0_, the more difficult it was to control the epidemic. When R_0_ $\le$ 1, there was a unique disease-free equilibrium, and the disease-free equilibrium was globally asymptotically stable and the disease will gradually disappear. Finding out the disease-free equilibrium was of great significance to control the spread of disease. In this study, if the right end of the Eqs. (1–4) was zero ($n-\beta SI-uS=0,\beta SI-\omega E-uE=0,\omega E-\gamma I-I\left(u+p\right)=0,\gamma I-uR=0$), and E (t) = 0, I (t) = 0, the disease-free equilibrium point $\mathrm{P}0=\left(\frac{n}{u},\mathrm{0,0},0\right)$ can be obtained. Using the method of spectral radius to calculate the basic reproduction number ${R}_{0}=\rho \left(F{V}^{-1}\right)$, where the basic reproducing number (R_0_) was equal to the spectral radius of the matrix ρ(*FV*^−1^), and R_0_ was the largest eigenvalue of the matrix (*FV*^−1^). The matrices, F and V, were given by,

5 $$\mathrm{F}=\left[\begin{array}{c}\beta SI\\ 0\\ 0\\ 0\end{array}\right],$$

and,

6 $$\mathrm{V}=\left[\begin{array}{c}\left(w+u\right)E\\ \left(r+u+p\right)I-\omega E\\ \beta SI-us-n\\ uR-rI\end{array}\right],$$

Seeking partial derivation:

7 $$\mathrm F=\begin{bmatrix}0&\beta S\\0&0\end{bmatrix},\;\mathrm V=\begin{bmatrix}(\omega+u)&0\\-\omega&(\gamma+u+p)\end{bmatrix},$$

Inverse matrix solution:

8 $${V}^{-1}=\left[\begin{array}{cc}\frac{1}{(\omega +u)}& 0\\ \frac{1}{(\omega +u)(\gamma +u+p)}& \frac{1}{(\gamma +u+p)}\end{array}\right],$$

9 $${FV}^{-1}=\left[\begin{array}{cc}0& \beta S\\ 0& 0\end{array}\right]\times \left[\begin{array}{cc}\frac{1}{(\omega +u)}& 0\\ \frac{1}{(\omega +u)(\gamma +u+p)}& \frac{1}{(\gamma +u+p)}\end{array}\right]=\left[\begin{array}{cc}\frac{\beta S\omega }{(\omega +u)(\gamma +u+p)}& \frac{\beta S}{(\gamma +u+p)}\\ 0& 0\end{array}\right],$$

The largest eigenvalue was $\frac{\beta S\omega }{\left(\omega +u\right)\left(\gamma +u+p\right)}$, $\mathrm{S}=\frac{n}{u}$, so in this case, R_0_ was given by

10 $${R}_{0}=\frac{\beta n\omega }{u(u+\omega )(\gamma +u+p)}$$

### Scenario tree quantitative risk analysis

Quantitative risk analysis (QRA) is a systematic analysis method, which integrates quantitative information about events and is used as a tool to systematically assess the possibility of consequential events. The commonly used scenario tree quantitative model is composed of a series of events. According to the probability of each event, after calculation, it is used to predict the uncertainty of future events.

The quantitative risk assessment model was established to assess the risk of PPRV infection in live sheep exported from India. The probability distribution of each node determines the overall output risk. For the distribution of each node, we can use the formula to calculate, refer to the relevant literature, or collect the annual data for data fitting in @ Risk. In data fitting, according to the Akaike information criterion (AIC), the distribution type closest to the actual input item is selected. Based on the sensitivity analysis of the Spearman correlation coefficient, the rank correlation coefficient between the output variable and input distribution was analyzed. The higher the correlation, the greater the impact on the model output. The model was developed in an Excel spreadsheet (Microsoft Office Excel, 2016) using @ Risk 7.6.1 (Palisade Corporation). Run 10,000 iterations for Monte Carlo sampling.

**Fig. 10:**
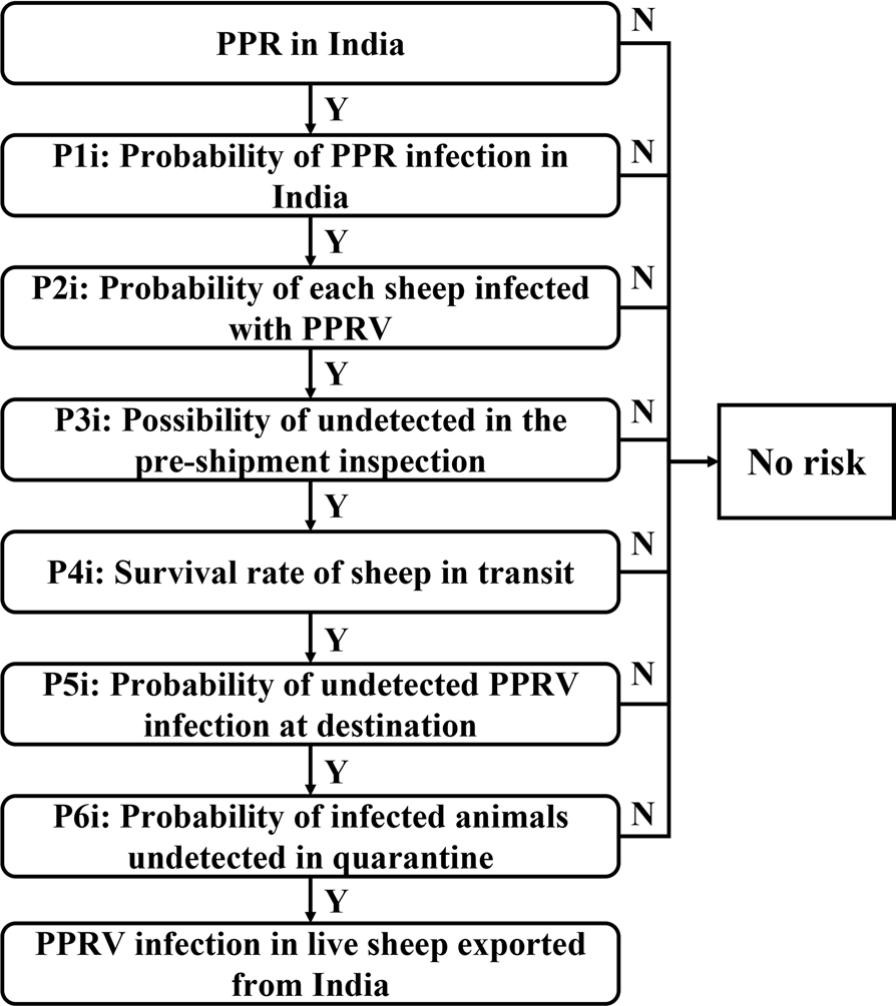
Scenario tree of releasing PPRV from live sheep exported from India

In this study, a scenario tree quantitative risk model of PPR virus in India through live sheep trade was constructed (Fig. 10). The probability distribution of each node in the scenario tree was shown as follows and summarized in Table 5.

#### P1i: Probability of PPR infection in India

According to WOAH statistics, there were 1191 outbreaks of PPR in India from 2010 to 2018. The probability of an outbreak of PPR at least once a month in India:

11 $$\mathrm{P}1\mathrm{m}=1-{\mathrm{e}}^{\left(-\mathrm{t}\times\uplambda \right)}$$

where t was the time interval, t = 1 (for one month). According to the outbreak data from, the average number of PPR outbreaks per month was calculated λ = a/b, a was the total number of PPR outbreaks in India; b was the total number of months during the period of statistical outbreak data.

#### P2i: Probability of each sheep infected with PPRV

(P2ia + P2ib)/2 was used to calculate the Probability of each sheep and goats infected with PPRV, where P2ia was the prevalence of sheep, and P2ib was the prevalence of goats.

#### P3i: Possibility of undetected in the pre-shipment inspection

Live sheep need to be detected before shipment, and the common detection method was ELISA. According to the literature, the sensitivity of ELISA was uniform (0.8728, 0.9703). Therefore, we used 1-uniform (0.8728, 0.9703) to indicate the probability that undetected the infected live sheep.

#### P4i: Survival rate of sheep in transit

The mortality of sheep in transit was pert (0.0013, 0.0086, 0.0553), therefore, 1- pert (0.0013, 0.0086, 0.0553) was used to represent the survival rate of sheep in transit.

#### P5i: Probability of undetected PPRV infection at destination

We used uniform distribution (0.35, 0.6) to indicate that infected sheep will die during transportation. The survival probability of sheep was 1-uniform (0.35, 0.6). When detected by ELISA, the probability of undetected infected sheep was 1-uniform (0.8728, 0.9703). Therefore, probability of undetected PPRV infection at destination was (1-uniform (0.8728, 0.9703)) × (1-Uniform (0.35, 0.6)).

#### P6i: Probability of infected animals undetected in quarantine

After arriving at the destination, the infected animals contact susceptible animals, leading to the spread and infection of PPRV. The quarantine can effectively detect infected animals. In this paper, beta (42.11, 1.83) was used to indicate the possibility of quarantine, and beta (15.03, 2.55) was used to indicate the possibility of detection during quarantine. Therefore, the probability of infected animals undetected in quarantine was 1-beta (42.11, 1.83) × beta (15.03, 2.55).

The output risk was the Probability of PPRV infection in live sheep exported from India (Pi):

12 $$\mathrm{Pi}=\mathrm{P}1\mathrm{i}\times \mathrm{P}2\mathrm{i}\times \mathrm{P}3\mathrm{i}\times \mathrm{P}4\mathrm{i}\times \mathrm{P}5\mathrm{i}\times \mathrm{P}6\mathrm{i}$$

The probability of at least one sheep infected with PPRV exported from India (Pq) was $\mathrm{Pq}=1-{(1-\mathrm{Pi})}^{\mathrm{q}}$, where q was the export volume of live sheep in India, according to the data of FAO, the distribution was triang (147,649, 147,756,935,999). Binomial (q, Pi) distribution was used to estimate the number of infected sheep exported per year.

## Supplementary Information

**Additional file 1: S1 Fig.** Time series curve. Note A: outbreak time series; B: seasonal decomposition curve. **Table S1.** Comparison of time series modeling results. **Table S2.** Prediction of the two time series models.

## Data Availability

The datasets used and/or analyzed during the current study are available from the corresponding author on reasonable request. Data used in the present study was obtained from the global animal disease information system (http://empres-i.fao.org/eipws3g/#h=0), World Organization for Animal Health (https://www.woah.org/), the Food and Agriculture Organization (http://www.fao.org/) and National Bureau of statistics of India (https://www.mospi.gov.in/).
