# Supplementary material for: Epidemiologic and import risk analysis of Peste des petits ruminants between 2010 and 2018 in India
Source: BMC Vet Res. 2022 Nov 29;18:419. doi: 10.1186/s12917-022-03507-x (PMC9707066; doi:10.1186/s12917-022-03507-x)
Supplement: Supplementary file 1 — Additional file 1: S1 Fig. Time series curve. Note A: outbreak time series; B: seasonal decomposition curve. Table S1. Comparison of time series modeling results. Table S2. Prediction of the two time series models. [file 12917_2022_3507_MOESM1_ESM.docx]

**Additional file 1**


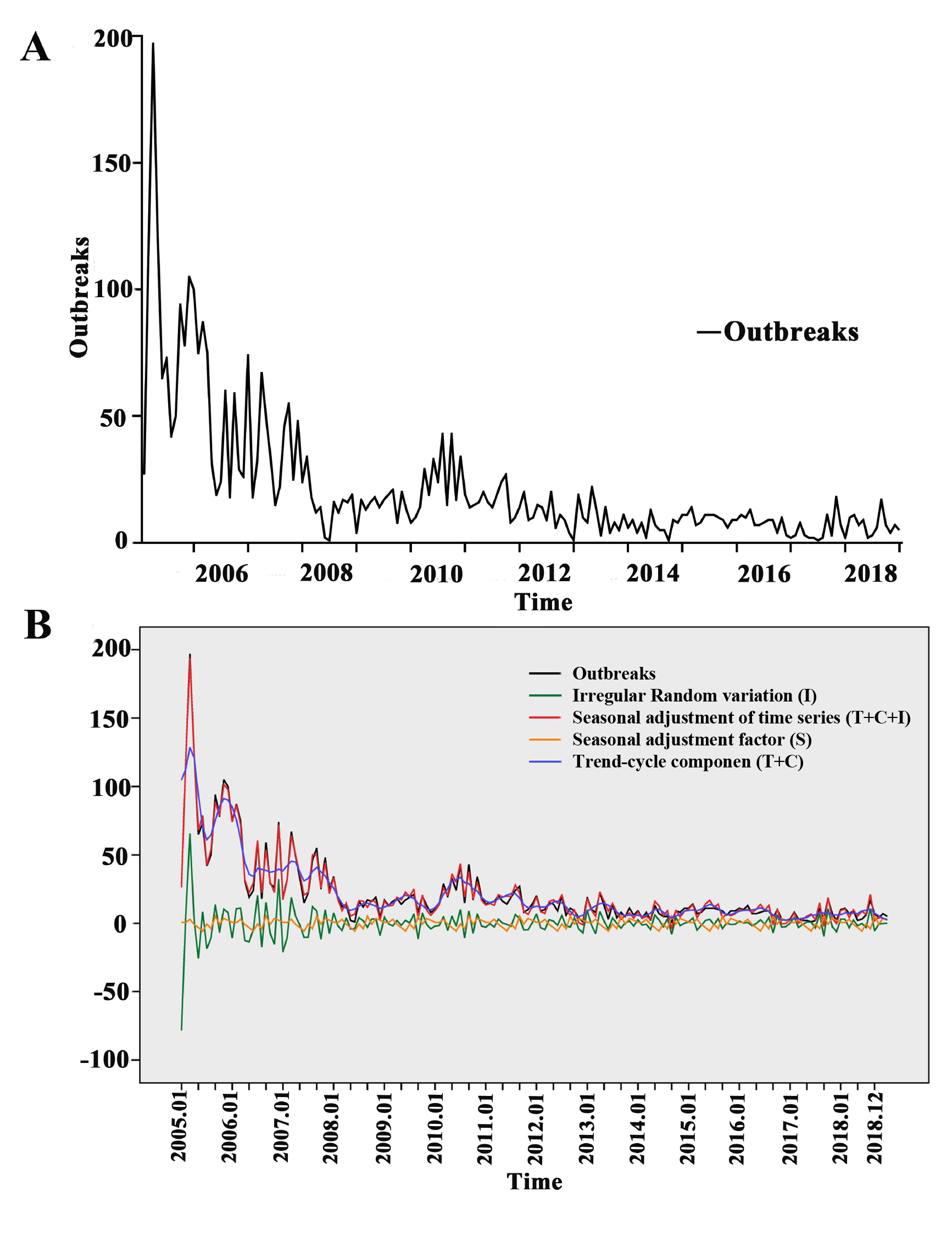


**S1 Fig. Time series curve.** Note A: outbreak time series; B: seasonal decomposition curve

| **Table S1. Comparison of time series modeling results** | | | | | | |
| --- | --- | --- | --- | --- | --- | --- |
| Model | Stationary R^2^ | R^2^ | RMSE | BIC | *P* | White noise sequence |
| Winters multiplication | 0.704 | 0.75 | 13.5 | 5.297 | 0 | No |
| ARIMA(2,0,1)(0,0,0) | 0.652 | 0.593 | 17.271 | 5.82 | 0.106 | Yes |
| Winters addition | 0.527 | 0.651 | 15.93 | 5.628 | 0.021 | No |
| ARIMA(2,1,1)(0,0,0) | 0.158 | 0.609 | 16.968 | 5.785 | 0.011 | No |
| ARIMA(1,0,1)(0,0,0) | 0.645 | 0.566 | 17.776 | 5.847 | 0.009 | No |
| Note RMSE: Root Mean Squared Error; BIC: Bayesian Information Criterion | | | | | | |

| **Table S2. Prediction of the two time series models** | | | | | | |
| --- | --- | --- | --- | --- | --- | --- |
| Time | Winters multiplication | | | ARIMA(2,0,1)(0,0,0) | | |
|  | Predictive value | LCL | UCL | Predictive value | LCL | UCL |
| 2020.01 | 6 | -25 | 37 | 11 | 0 | 38 |
| 2020.02 | 9 | -27 | 45 | 11 | 0 | 39 |
| 2020.03 | 12 | -32 | 56 | 11 | 0 | 39 |
| 2020.04 | 8 | -28 | 45 | 11 | 0 | 40 |
| 2020.05 | 6 | -26 | 38 | 11 | 1 | 41 |
| 2020.06 | 5 | -27 | 37 | 11 | 1 | 42 |
| 2020.07 | 7 | -29 | 42 | 12 | 1 | 42 |
| 2020.08 | 5 | -28 | 39 | 12 | 1 | 43 |
| 2020.09 | 9 | -35 | 53 | 12 | 1 | 44 |
| 2020.1 | 6 | -31 | 43 | 12 | 1 | 45 |
| 2020.11 | 8 | -36 | 52 | 12 | 1 | 45 |
| 2020.12 | 8 | -36 | 52 | 12 | 1 | 46 |
| Note LCL: Lower confidence limit; UCL: Upper confidence limit | | | | | |  |
